# Satisfaction of patients fit with a hearing aid in a high complexity clinic

**DOI:** 10.5935/1808-8694.20120011

**Published:** 2015-11-20

**Authors:** Fernanda Soares Aurélio, Simone Pereira da Silva, Liliane Barbosa Rodrigues, Isabel Cristiane Kuniyoshi, Marília Silva e Nunes Botelho

**Affiliations:** a MSc in Human Communication Disorders - UFSM. Speech and Hearing Therapist - Threshold hearing rehabilitation and assessment clinic. Professor - Faculdade São Lucas, Porto Velho - RO; b BS; Speech and Hearing Therapist; c Specialist; Speech and Hearing Therapist - Policlínica Osvaldo Cruz. Professor - Faculdade São Lucas, Porto Velho - RO; d MSc; Speech and Hearing Therapist; Professor - Faculdade São Lucas, Porto Velho - RO; e Specialist; Speech and Hearing Specialist. Faculdade São Lucas

**Keywords:** hearing, hearing loss, patient satisfaction, quality of life, rehabilitation

## Abstract

The process of selecting and fitting hearing aid devices is only effective and only bring about good outcomes if the individual makes effective use of the device. Therefore, the individuals need to be happy with the outcome.

**Aim:**

To check the satisfaction of adults and elderly patients concerning their hearing aid in a high complex care clinic accredited by the Unified Health System, and to correlate this outcome with the variables related to age, gender, fitting period, daily use, as well as the type of sound amplifying device.

**Methods:**

This is a descriptive cross-sectional study in which 60 subjects were evaluated using the questionnaire: Satisfaction with Amplification in Daily Life, applied by means of oral presentation, in individual interviews by the researcher. This instrument is divided into subscales: positive effects, service and costs, negative factors and personal image.

**Results:**

It was shown that the subjects of this study were very happy with the use of hearing aid devices. There was significant difference in relating the daily use of the devices with the overall satisfaction score and subscale of personal image.

**Conclusion:**

It was found that the subjects of the study were very happy with the use of hearing aids, although satisfaction was not related to the variables: age, gender, time of use and device type. In general, participants with higher daily use are happier.

## INTRODUCTION

Hearing is a fundamental sense in life, it is the very basis of human communication. Hearing loss causes not only a handicap in the person's capacity to perceive sounds, it brings about psychosocial compromises, keeping the individual from having a healthy social life and playing his/her role in society, which has a great impact on the person's quality of life^1^.

Hearing loss has been considered a disabling disease for a long time. In recent years, much has been done to mitigate such stigmas and provide a better quality of life for hearing impaired individuals^2^.

The difficulties brought about by sensorial deprivation may be mitigated with the use of an Individual Sound Amplification Device (ISAD), which enables one to recover sound perception, reducing background noises, causing an improvement in communication skills^3^, the prognosis will depend on the site of the lesion, the degree of hearing loss and, especially, the expectations associated with the amplification^4^.

The goal of speech and hearing therapists who fit ISAD is to try to please the user, guaranteeing better communication possibilities and contributing to the patient's quality of life^5^. However, one of the greatest difficulties faced by these professionals is how to validate the success obtained from using such devices^6^.

Despite all the advantages technological progresses may bring about, it is mandatory to understand the real hearing difficulties of the users and their expectations as to the amplification, so as to better guide and individualize clinical care^2^.

Performance with the hearing aid reported by the user can better guide the healthcare professionals as to the proper actions to take, enabling one to recognize the advantages provided by these devices in relation to hearing difficulties, preventing users to give up on them, increasing daily use and, consequently, user satisfaction.

Therefore, in order to better fit an ISAD to obtain better patient satisfaction, the user's opinion is certainly one of the most important factors.

Today, questionnaires have been used with the aim of assessing user satisfaction and drawbacks in relation to ISAD use, and to know whether the fitting has been effective, providing for the auditory, social and emotional needs of these individuals.

One of the questionnaires created to assess user's satisfaction concerning the ISAD, as well as its effectiveness is the *Satisfaction with Amplification in Daily Life* (SADL) questionnaire, proposed by Cox & Alexander^7^.

To create this instrument, first they carried out structured interviews with ISAD users, aiming at obtaining the most important elements associated with satisfaction. Afterwards, experimental satisfaction items were created for each area of importance and, from this, they created a questionnaire with 25 items, which was distributed to ISAD users. They obtained and analyzed the results from 257 individuals, thus getting to the final questionnaire, which presented a high reliability in the test-retest correlation.

The SADL was validated^8^ in a sample of 196 individuals, from 13 private audiology clinics, where they confirmed its capacity to quantify patient satisfaction.

Moreover, studies state that such self-assessment instrument is hugely valuable and easy to apply, efficient and accurate in assessing the individual's satisfaction with the amplification device, encompassing numerous aspects^9^, ^10^, ^11^.

Most of the population from the State and region go through the selection and fitting of hearing aids process in a private high complexity clinic, which has a partnership with the public healthcare service - *Sistema Único de Saúde* (SUS), according to ordinance 589, from October 8, 2004^12^. This clinic is a reference clinic in hearing health in the State and serves the population in the capital, as well as the patients coming from other cities in the country side and neighboring states, carrying out about 35 fittings per month.

Based on the aforementioned, the present paper aimed at establishing the hearing satisfaction of adult and elderly patients fitted with hearing aids, in the aforementioned clinic and to correlate patient satisfaction with age, gender, time using the device and type of ISAD.

## METHOD

This is a descriptive cross-sectional study, approved by the Ethics Committee of the Institution, under protocol # 634/11. It was developed in a high complexity service which has a partnership with the Brazilian Public Healthcare System - *Sistema Único de Saúde* (SUS).

Inclusion criteria to participate in the study were: individuals from both genders, with ages higher than 18 years, with any type and degree of hearing loss, fitted with an ISAD - supplied by SUS, seen in the clinic where the study was carried out, who agreed to participate in the study and signed an Informed Consent Form.

We took off the study those individuals to whom ISADs were not indicated, those who refused to participate in the study, individuals younger than 18 years of age and those with difficulties understanding and expressing themselves concerning the questionnaire.

For the sample calculation, we used as basis the mean number of repairs done to devices supplied by the SUS to adults and elderly, by month in the study venue, which is around 155. Using a 10% error and a 95% confidence level, we came to a sample of 60 patients - number of individuals in the sample.

Among these 60, 33 were females and 27 were males, with ages varying between 18 and 91 years and mean age of 61 years, which came to the referral clinic between September 22 and October 25 of 2011.

Of the individuals in the sample, 49 (80%) used the behind-the-year ISAD and 11 (20%) had the intracanal one. All the individuals had sensorineural or mixed, bilateral hearing loss and used digital ISAD for a minimum time of two weeks - time during which the subjects came to the clinic for the first post-fitting check.

The user's satisfaction with the ISAD was assessed by means of the Brazilian Portuguese version of the *Satisfaction with Amplification in Daily Life* (SADL) questionnaire, made available by the authors^13^, and such instrument is considered efficient and accurate to assess the individual's satisfaction with the amplification^10^^,^^11^.

The questionnaire is broken down into four subscales, namely: positive effects, services and costs, negative factors and personal image. The mean score from the four subscales results in the global satisfaction score.

The questionnaire has 15 multiple-choice questions, with seven options of answer for each: nothing, a little, somehow, average, considerably, much and very much. The answers are equivalent to a 7-point scale, in which the lowest score is 1, corresponding to the “nothing” answer, and the highest value is 7, corresponding to the “very much” answer, pointing to, respectively, the lowest and the highest degrees of satisfaction. Questions numbers 2, 4, 7 and 13 correspond to the items called “reverse”, in which the score of 7 corresponds to the “nothing” and score 1 corresponds to the “very much” answer.

The participants were instructed to assign a score between one and seven for each question.

Besides the 15 questions, the subjects were asked about the time they spend using the device daily, with the following options for answers: none, less than one hour a day, from 1 to 4 hours a day, between 4 and 8 hours a day and between 8 and 16 hours a day.

The hearing loss type and degree, as well as the time using the ISAD were obtained by means of the study carried out from the patients' charts.

The questionnaire was deployed by the researcher, in an unbiased way, by means of a verbal presentation and individual interviews, in order to make sure the individuals in the sample understood the questions and, consequently, improving the quality of the data obtained.

The compiled data was analyzed by using the *Analysis of Variance* (ANOVA) and Pearson's correlation statistical tests, both with a 5% significance level (*p* ≤ 0.05). The variables assessed were: age, gender, time spent using the device daily, time since the first fitting and type of ISAD utilized.

In order to analyze the level of satisfaction, we used the standards proposed by the authors of the questionnaire^7^. The scores which remained below the 20^th^ percentile indicated patient dissatisfaction with the amplification device, scores between the 20^th^ and the 80^th^ percentiles indicated patient satisfaction and, finally, scores higher than 80 indicated that the subjects were very happy with the amplification device (Chart 1).

**Chart 1:** Mean values of the 20^th^ and 80^th^ percentiles for the global score and for each SADL subscale.

Score	Mean	20^th^ percentile	80^th^ percentile
Positive effects	4.9	3.8	6.1
Services and costs	4.7	4.0	5.7
Negative factors	3.6	2.3	5.0
Personal image	5.6	5.0	6.7
Global	4.9	4.3	5.6

SADL: *Satisfaction with Amplification in Daily Life.*

## RESULTS

Upon comparing the mean values by global scores and by the subscales found in this study with the values obtained from the study carried out by the authors of the questionnaire^7^, it was possible to check the satisfaction of the users in the questions regarding the negative factors and personal image subscales, since the mean values found were between the 20^th^ and the 80^th^ percentiles. In the questions associated with the positive effects and service subscales, as well as in the global score, we had a high degree of satisfaction, because the mean values were higher than those in the 80^th^ percentile (Chart 2). However, the satisfaction found was not correlated with the subject's age (Table 1).

**Chart 2:** Global and subscale scores from patients with hearing prosthesis in the Clínica Lumiar, assessed between September and October of 2011, and the questionnaire's normalization^7^.

Positive Effects	6.2	4.9 (3.8-6.1)
Services and Costs	6.1	4.7 (4.0-5.7)
Negative Factors	4.9	3.6 (2.3-5.0)
Personal image	6.2	5.6 (5.0-6.7)
Global	5.9	4.9 (4.3-5.6)

**Table 1:** Correlation between age and global and subscale satisfaction score of the patients fit with a prosthesis in the Clínica Lumiar and assessed between September and October of 2011.

Age	Correlation	*p*-value
Positive Effects	13.6%	0.298
Services and Costs	19.7%	0.132
Negative Factors	5.3%	0.686
Personal Image	14.3%	0.277
Global	17.9%	0.171

Statistical test: Pearson's correlation; significance level *p*≤ 0.05.

%: percentage.

Statistical test: Pearson's correlation; significance level *p* ≤ 0.05. %: percentage.

There was no significant difference also comparing the gender and the global satisfaction, and by subscale (Table 2).

**Table 2:** Comparing the global and subscale satisfaction score according to gender, in patients fit with a prosthesis in the Clínica Lumiar, assessed between September and October of 2011.

	Gender	Mean	Median	Standard Deviation	Lowest	Highest	n	CI	*p*-value
	Females	36.8	37	4.1	26	42	33	1.4	
Positive Effects	Males	37.0	37	3.5	30	42	27	1.3	0.782
	Females	18.6	19	2.3	13	21	33	0.8	
Services and Costs	Males	18.0	18	2.4	12	21	27	0.9	0.302
	Females	14.2	13	3.6	8	21	33	1.2	0.218
	Males	15.4	15	3.7	7	21	27	1.4	
	Females	18.6	19	2.6	12	21	33	0.9	0.728
	Males	18.4	18	2.5	14	21	27	0.9	
	Females	88.2	88	9.1	65	104	33	3.1	
Global	Males	88.8	89	8.3	70	105	27	3.1	0.793

Statistical test: ANOVA; significance level *p*≤ 0.05. n: number of subjects; CI: Confidence Interval.

Upon associating the daily use of the devices with the global satisfaction and by subscales, we found a significant difference in the global score and in the personal image subscale (Table 3).

**Table 3:** Correlation between the daily use of an ISAD (individual sound amplification device) and global and subscale satisfaction scores in the Clínica Lumiar, assessed between September and October of 2011.

	Daily use	Mean	Median	Standard deviation	Lowest	Highest	n	CI	*p*-value
	Less than 1h/day	34.0	34	1.4	33	35	2	2.0	
Positive Effects	From 1 to 4h/day	34.0	35	2.8	30	36	4	2.8	0.051^#^
	From 4 to 8h/day	34.9	35.4	3.6	30	40	10	2.2	
	From 8 to 16h/day	37.7	39	3.8	26	42	44	1.1	
	Less than 1h/day	20.5	20.5	0.7	20	21	2	1.0	
Services and costs	From 1 to 4h/day	16.5	17.5	2.4	13	18	4	2.3	0.056^#^
	From 4 to 8h/day	17.0	16.5	2.2	14	21	10	1.3	
	From 8 to 16h/day	18.7	19	2.3	12	21	44	0.7	
	Less than 1h/day	16.0	16	7.1	11	21	2	9.8	
Negative Factors	From 1 to 4h/day	15.5	16	5.2	9	21	4	5.1	0.294
	From 4 to 8h/day	12.7	13.5	3.3	7	19	10	2.0	
	From 8 to 16h/day	15.0	14.5	3.5	8	21	44	1.0	
	Less than 1h/day	20.5	20.5	0.7	20	21	2	1.0	
Personal Image	From 1 to 4h/day	19.5	20	1.7	17	21	4	1.7	0.017*
	From 4 to 8h/day	16.4	16	2.5	13	20	10	1.6	
	From 8 to 16h/day	18.8	19	2.4	12	21	44	0.7	
	Less than 1h/day	91.0	91	8.5	85	97	2	11.8	
Global	From 1 to 4h/day	85.5	84.5	7.3	78	95	4	7.2	0.014*
	From 4 to 8h/day	81.0	77	8.0	70	96	10	5.0	
	From 8 to 16h/day	90.3	90.5	8.1	65	105	44	2.4	

* Significance level (*p* ≤ 0.05) - ANOVA statistical test. # tending to significance. ISAD: individual sound amplification device; n: number of subjects; CI: Confidence Interval.

Upon studying the findings concerning global satisfaction and the personal image subscale, according with the time of daily use, in pairs, we found a significant difference comparing the subjects who reported using the ISAD between 8 and 16 hours per day and the findings from those who reported using it between four and eight hours daily (*p* = 0.009 and *p* = 0.023), and we found a higher mean value among those individuals who used the device between eight and sixteen hours per day.

Regarding the comparison of the global satisfaction degree and by subscale with the fitting time variable, in which the subjects were distributed in four groups (less than six weeks, from six weeks to 11 months, from one to ten years and for more than ten years), we also did not find significant differences (global: *p* = 0.996; positive effects: *p* = 0.982; services and costs: *p* = 0.393; negative factors: *p* = 0.815; personal image: *p* = 0.924).

We did not find statistically significant differences upon comparing the global satisfaction and subscale among users of behind-the-year and intracanal ISADs (Table 4).

**Table 4:** Correlation between the type of ISAD and the global and subscale satisfaction scores of patients fit with a hearing aid in the Clínica Lumiar between September and October of 2011.

	Type of ISAD	Mean	Median	Standard Deviation	Lowest	Highest	n	CI	*p*-value
	Intracanal	36.6	37	3.2	32	41	11	1.9	
Positive effects	Behind-the-ear	36.9	37	4.0	26	42	49	1.1	0.816
	Intracanal	18.0	18	2.0	15	21	11	1.2	
Services and costs	Behind-the-ear	18.4	19	2.5	12	21	49	0.7	0.630
	Intracanal	15.5	14	4.3	9	21	11	2.6	
Negative Factors	Behind-the-ear	14.6	14	3.6	7	21	49	1.0	0.468
	Intracanal	18.0	19	2.8	14	21	11	1.6	
Personal Image	Behind-the-ear	18.7	19	2.5	12	21	49	0.7	0.440
	Intracanal	88.1	90	8.5	76	98	11	5.0	
Global	Behind-the-ear	88.5	88	8.7	65	105	49	2.4	0.880

Statistical test: ANOVA; level of significance *p* ≤ 0.05. ISAD: Individual Sound Amplification Device; n: number of subjects; CI: Confidence Interval.

Statistical test: ANOVA; significance level *p* ≤ 0.05. n: number of subjects; CI: Confidence Interval.

## DISCUSSION

After using the SADL we could notice that, in general, the subjects of this study were very happy with their ISADs, having a global mean value of 5.9, value above the 80^th^ percentile of the original study, ratifying what was found in other studies^1^^,^^14^. Moreover, we could notice that this finding was higher than others published in the literature^15^^,^^16^.

Concerning the scores of the individuals in the positive effects subscale, in this study we found a value higher than the ones advocated by the questionnaire authors^7^, as well as, when comparing to other Brazilian studies^15^^,^^16^, which shows that the participants were very happy with their ISADs in this subscale, which checks the sound quality and the improvement in communication.

In the services and costs subscale, in which we encompass the services of hearing rehabilitation and the hearing aid fitting cost, this study found a mean value of 6.1, which was higher than the 80^th^ percentile, suggesting much satisfaction and which, if one compares the mean value found by the authors of the SADL^7^ (4.7) and other studies which used the same instrument^1^^,^^15^, with mean values of 6.0 and 5.6, resulting in a higher score. The subjects of this study were benefited by the ISAD supplied by the SUS, which, possibly, together with the satisfaction with the service rendered, helped raise this mean value, reflecting the high degree of satisfaction vis-à-vis the items associated to the services and cost subscale.

The negative factors subscale had a mean value of 4.9, a result which was lower than what was seen in other studies considered^1^^,^^16^, which found mean values of 5.0 and 5.2; however, higher than what was found in a study carried out with ISAD users in the state of Tocantins, which found a mean value of 4.18^15^ and higher than the one found by the authors of the questionnaire^7^, who found a mean value of 3.6. This was the subscale with the lowest score, for it assesses aspects considered problematic in fitting, such as performance in a noisy background, feedback and the use of telephone. Even then, the subjects assessed in the present study obtained a mean value between the 20^th^ and the 80^th^ percentiles^7^, indicating patient satisfaction.

The subscale which assesses personal image had a mean value of 6.2, higher than what was found in other studies^1^^,^^15^, ^16^, ^17^, with mean values of: 5.5, 5.3, 5.6 and 5.7. This value is also higher than the mean reported in the original study^7^, indicating patient satisfaction as far as personal image is concerned and the stigma associated with using a hearing aid.

We can notice that in the sample studied, there was no relationship between age and satisfaction, matching findings from another study^9^, which concluded that younger individuals were happier. This difference happened because the study in question was carried out only with users of the intracanal devices, which cosmetically pleases the users and makes it harder for the elderly to handle them.

This study did not show differences in the degree of satisfaction among the genders, corroborating what has been published in the literature^9^, which reports equal satisfaction rates between men and women. Until very recently, men were more resistant to wearing hearing aids and were less happy with them, maybe because of aesthetic issues. Moreover, most women carry out daily activities which require more communication than men, causing greater resistance among men^18^, contrary to the present study, in which we state the concern from both genders considering their well-being and performance in society.

Most individuals reported using the hearing aid for more than 8 hours, in agreement with reports from other studies^1^^,^^19^ which shows that users use sound amplification for more than eight hours a day, a considerable amount of time, since from the 24 hours a day, the subjects sleep for approximately 8 hours, thus leaving only 16 hours of which 8 are spent using a hearing aid by the majority, showing that the hearing aid is an integral part of the day-to-day of these individuals^1^. The current study also showed that the individuals who used ISADs for longer were happier than expected, because the longer the use, the better their adaptation concerning this technology.

Upon correlating the daily use of hearing aids with the patient satisfaction per subscale, there was no significant difference between the variable mentioned and the positive effects and services and cost subscales; however, results tend to significance (*p* = 0.051; *p* = 0.056), suggesting that if this study's sample were bigger, the results could have been significant.

We found no relationship between satisfaction and time of use. Studies found in the literature report that the ISAD reintroduces the hearing stimulation after amplification, causing a neural plasticity which enables the central pathways to reorganize and start producing positive effects on hearing skills^20^. The improvement in hearing brought about by the stimulation is known as acclimatization phenomenon and may happen by three months after fitting the hearing aid^21^, from six to 12 weeks after using the amplification^22^ and, according to some authors, after the second month of use^23^. In this study, individuals with less than six months of use with those with more than ten years were happy, which leads us to believe that acclimatization may happen in less than three months, and it is inversely proportional to the time of use, in other words, the longer the time of daily use, the lower is the necessary time for the individual to get used to the device.

The last two findings are complementary, having seen that, since the participants of the study used their devices for over 8-hours a day, adaptation happened in less time.

We did not find any difference in the degree of satisfaction among the behindthe-ear and intracanal ISAD users, and such finding is not in agreement with what has been found in other studies^14^, which found a greater satisfaction among users of the intracanal ISAD. Another study carried out in one institution of the state of Tocantins, also found a high index of satisfaction among the users of behind-the-ear ISAD, as far as personal image is concerned^15^. The present study did not find dissatisfaction associated with the device model, a finding which indicates that the users are more concerned with their well-being, increasingly enhancing quality of life and participation in society than aesthetic factors.

Finally, we noticed that the SADL questionnaire proved to be an efficient and accurate instrument to assess the level of satisfaction of hearing aid users, as per published in the literature^10^, ^11^.

**Table:** Questionnaire. Satisfaction with the hearing device in your daily life. Portuguese version proposed by the authors (Cox and Alexander, 1999).

Name:____________________________________	
Date of birth __/__/____Date__/__/____	
A Nothing	
B A little	
C More or less	
D Average	
E Considerably	
F Much	
G Very Much	
1. Do your hearing aids help you understand what those who most frequently talk to you say, when compared to not using the devices?	A B C D E F G
2. Do you feel frustrated when your hearing aid captures sounds which do not allow you to hear what you would like to hear?	A B C D E F G
3. Are you convinced that having acquired your hearing aids was your best option?	A B C D E F G
4. Do you think people better perceive you have a hearing loss when you are using your hearing aids?	A B C D E F G
5. Do your hearing aids reduce the number of times you have to ask people to repeat what they have just said?	A B C D E F G
6. Do you think your device compensates for your problem?	A B C D E F G
7. Are you unhappy for not achieving the volume you want without the device beeping?	A B C D E F G
8. How happy are you with the looks of your device?	A B C D E F G
9. Does using the device improve your self-confidence?	A B C D E F G
10. How natural is the sound you get from your device?	A B C D E F G
11. How much do your devices help you talk on telephones which do not have volume amplification? (If you can hear well on the phone without the devices, tick here O)	A B C D E F G
12. How competent was the person who provided you with the devices?	A B C D E F G
13. Do you think using the device makes you feel less able?	A B C D E F G
14. Does the cost of your hearing aids seem reasonable?	A B C D E F G
15. Are you happy with the quality of your device (concerning the number of times it needed repairs	A B C D E F G
Experience with the current devices: ˆ Less than 6 weeks ˆ From 6 weeks to 11 months ˆ From 1 to 10 years ˆ More than 10 years	
All the experiences with the device (including the older and the current ones).	
Continues in Questionnaire. ˆ Less than 6 weeks ˆ From 6 weeks and 11 months ˆ From 1 to 10 years ˆ For more than 10 years	
Daily use of the devices: ˆ None ˆ Less than one hour per day ˆ From 1 to 4 hours per day ˆ From 4 to 8 hours per day ˆ From 8 to 16 hours per day	
Level of hearing difficulty (without the hearing device) ˆ None ˆ Average ˆ Moderate ˆ Moderate/Severe ˆ Severe	
For exclusive use of the audiologist	
Fitting the device	
Right Ear	Left Ear
Brand	Brand
Model	Model
Serial #	Serial #
Date of fitting	Date of fitting
Type: CIC, ITC, ITE, BTE	Type: CIC, ITC, ITE, BTE
Date of first fitting__/__/____	

## CONCLUSION

We noticed that the participants of the study are very happy with their ISADs; however, satisfaction has no relationship with the variables: age, gender, time of use, and type of individual sound amplification device. In general, the subjects with the longer time of daily use are happier with their devices.
